# A Study on Pre-Oxidation of Petroleum Pitch-Based Activated Carbons for Electric Double-Layer Capacitors

**DOI:** 10.3390/molecules27103241

**Published:** 2022-05-18

**Authors:** Jong-Woo Kim, Dae-Won Kim, Seul-Yi Lee, Soo-Jin Park

**Affiliations:** Department of Chemistry, Inha University, 100 Inharo, Incheon 22212, Korea; whddn4451@naver.com (J.-W.K.); 22211399a@inha.edu (D.-W.K.)

**Keywords:** pre-oxidation, petroleum pitch-based activated carbons, supercapacitors, electric double-layer capacitors (EDLCs), microporous

## Abstract

Electric double-layer capacitors (EDLCs) are an excellent electrochemical energy storage system (ESS) because of their superior power density, faster charge–discharge ability, and longer cycle life compared to those of other EES systems. Activated carbons (ACs) have been mainly used as the electrode materials for EDLCs because of their high specific surface area, superior chemical stability, and low cost. Petroleum pitch (PP) is a graphitizable carbon that is a promising precursor for ACs because of its high carbon content, which is obtained as an abundant by-product during the distillation of petroleum. However, the processibility of PP is poor because of its stable structure. In this study, pre-oxidized PP-derived AC (OPP-AC) was prepared to investigate the effects of pre-oxidation on the electrochemical behaviors of PP. The specific surface area and pore size distribution of OPP-AC were lower and narrower, respectively, compared to the textural properties of untreated PP-derived AC (PP-AC). On the other hand, the specific capacitance of OPP-AC was 25% higher than that of PP-AC. These results revealed that pre-oxidation of PP induces a highly developed micropore structure of ACs, resulting in improved electrochemical performance.

## 1. Introduction

With the sharply increasing human population and the life expectancies with advanced technology, energy shortages and global warming are becoming serious issues [1]. The identification of renewable energy sources and the development of efficient energy storage devices for low-carbon and sustainable economic development have become important [2,3,4,5]. Electrochemical energy storage and conversion systems, such as fuel cells, metal/air batteries, supercapacitors, etc., have attracted great attention as promising technologies to address the rapid energy demands and environmental concerns [6].

Electric double-layer capacitors (EDLCs) have been regarded as the most important energy storage devices with potentially excellent power densities, safeties, and specific capacitances, as well as fast charge–discharge rates and long-cycle lives for practical applications [7,8]. Since the 19th century, when von Helmholtz [9] first proposed the concept and model of double layers, EDLCs have been extensively utilized for various applications, including uninterruptible power supplies and memory backup systems [10]. However, their low energy density limits their adoption in a wider range of applications. The energy storage behaviors of EDLCs are mainly limited by the accumulation of electronic and ionic charges at the interface between the electrode and electrolyte; therefore, it is well-documented that a large specific surface area of the electrodes is crucial to obtaining a high specific capacitance [11,12].

Recently, several researchers have reported that the pore size distribution also plays an important factor in determining the excellent electrode materials for EDLCs. Qu et al. have discussed in detail the relationship between the intrinsic pore size distribution and their electrochemical performance for EDLCs [13]. Chmiola et al. also reported that energy storage capacitance was increased abnormally in non-aqueous solvents when the pore size was less than 1 nm [14]. This result was attributed to the high level of ionic motion and the reduced dielectric constant in pores smaller than the size of their solvated shell under the action of potential. When the ions are compressed through the pores, the solvated shell is highly distorted, which brings the ion center closer to the electrode surface, thus leading to increased capacitance.

To achieve high specific capacitances, many researchers have therefore focused on various carbonaceous materials, such as carbon nanotubes (CNTs), graphene, carbon sphere, and activated carbons (ACs), which have been known to have a large specific surface area and high porosity [15,16,17]. CNTs-based supercapacitors are relatively lower than that of amorphous carbons or porous carbons [18]. The surface of CNTs has some impurities that are hard to be removed and have difficulty to dispersion in solvents due to high van der Waals forces. Thus, CNTs can achieve high power density and high specific capacitance by means of additional treatments, including a multiple-step purification using a mixed strong acid and harsh oxidation, but the processes are quite expensive, and the long-term stability of such materials has not been reached so far. These results have restricted the large-scale applications of EDLCs, and the accompanied numerous environmental pollutions are also serious matters to be considered. In the case of graphene, graphene has been regarded as the most promising candidate for the electrode materials for EDLCs since it has high electrical conductivity and a high theoretical specific surface area of over 2600 m^2^ g^−1^. However, the specific surface area of graphene is usually much lower than the theoretical one, and above all things, there is a big challenge in solving the problems for the commercialization of adequate EDLCs [19].

Among the various carbonaceous materials for EDLC electrodes, ACs have generally been the choice for producing the electrodes because of advantages such as a large specific surface area, stable physical and chemical properties, good electrical conductivity, and low cost. To prepare excellent porous ACs with high micropore volume and specific surface area, many attempts have recently been made via activation processes, including physical and chemical activation. In physical activation, suitable oxidizing gasifying agents (O_2_, CO_2_, and H_2_O) are employed to produce porosity, generally at a high temperature. Physical activation is an inexpensive and simplified method, but ACs were produced with low specific surface area, resulting in low capacitances. On the other hand, in chemical activation, carbonaceous materials are mixed with chemical agents (NaOH, KOH, ZnCl_2_, and H_3_PO_4_). KOH is most widely used due to the highest specific surface area that can be obtained compared to other chemical agents. Although both activation processes develop the porosity of carbon materials, forming small mesopores and micropores, chemical activation exhibits excellent performance in terms of developing porosity. In addition, it requires a lower activation temperature and shorter activation time than physical activation.

Carbon source as a precursor of ACs was a consideration factor for high specific surface area and excellent electrochemical performance. Various raw materials have been used as precursors to obtain ACs, including biomass, plants, and polymers [20,21,22]. Pitch, a by-product of coal cracking or crude oil distillation industry, is regarded as one of the most promising precursors for ACs because of its low-ash, high-carbon yields and easily graphitizable feature. Therefore, pitch-based ACs offer numerous advantages over other raw carbonaceous materials.

Petroleum pitch (PP), as one of the pitch-based carbons, is considered a promising precursor candidate because of its high carbon content, low price, as well as different characteristic structures and properties [23,24,25,26,27]. However, PP is limited in exhibiting high specific surface area and microporosity because of its stable micro-graphite structure, low softening point, and hard-to-create micropores due to their structural firmness [28]. Therefore, PP requires additional treatments to be used as an ACs precursor, including pre-heat treatments, solvent extractions, and melt filtrations [29,30,31,32]. Surface oxidation is another simple strategy to overcome the limitations of PP. Wu et al. synthesized mesoporous carbon from rice husks for supercapacitors using the pre-oxidation method in the air [33]. This result showed that their specific capacitance reached 176 F g^−1^ at a current density of 50 mA g^−1^, with a high specific surface area of 2009 m^2^ g^−1^. Xing et al. have also examined the effects of pre-oxidizing ACs in air, demonstrating that the pre-oxidation of ACs accelerates the decomposition of aromatic structures during activation, resulting in the formation of abundant oxygen-containing groups on the carbon surface [34]. Kierzek et al. summarized a variety of ACs as electrode materials for EDLCs [35]. Many research studies of EDLCs have been conducted using various carbon sources. However, few reports have described the use of pre-oxidized PP-derived ACs for EDLCs.

In this study, pre-oxidized PP-derived activation carbon (OPP-AC) was successfully prepared by oxidation at 300 °C in oxygen and subsequent chemical activation to obtain highly developed microporous carbons. The elemental compositions, surface chemical state, crystalline structural properties, surface morphologies, textural properties, and electrochemical performances were investigated by characterizing the prepared samples using elemental analysis, X-ray photoelectron spectroscopy (XPS), X-ray diffraction (XRD), Raman spectroscopy, scanning electron microscopy (SEM), N_2_ adsorption–desorption measurement, and galvanostatic measurement, respectively. The experimental data revealed that OPP-AC exhibits a higher (357 F g^−1^) specific capacitance than untreated PP-based AC (PP-AC) (285 F g^−1^), whereas the specific surface area of OPP-AC (1246 m^2^ g^−1^) was lower than that of PP-AC (1327 m^2^ g^−1^). The experimental data obtained in this study suggested that pre-oxidation facilitates the development of micropores in the ACs, which can be critical to forming nano-channels for rapid penetration of the electrolyte ions into the porous structure, leading to improved electrochemical performance.

## 2. Materials and Methods

### 2.1. Preparation of Precursor Sample

Petroleum pitch (PP) procured from Carbonix Co., Korea, and pre-oxidized petroleum pitch (OPP) were both used as precursors to prepare ACs. The OPP sample was prepared by the pre-oxidation of PP at 300 °C for 2 h at a heating rate of 5 °C min^−1^ under the flow of oxygen (200 cc min^−1^).

### 2.2. Preparation of Activated Carbons

First, the KOH impregnation was initiated by adding 2 g of PP (or OPP) to a 50 mL ethanol solution containing 6 g of KOH. The mixture was stirred at room temperature for 5 h and then dried at 110 °C for 24 h. The KOH-impregnated PP (or OPP) was heated to 800 °C at 5° C min^−1^ in an electrical furnace in a nitrogen atmosphere. After being held for 2 h at 800 °C and naturally cooled, the resulting mixture was neutralized in 1 M HCl and washed with distilled water until neutralization. Finally, the pitch-based ACs were dried at 110 °C in a vacuum oven for 24 h. The prepared non-oxidized and pre-oxidized ACs are denoted as PP-AC and OPP-AC, respectively.

### 2.3. Characterization

Elemental analysis was conducted to investigate the elemental compositions of samples using a FLASH EA 1112 (Thermo Electron Co., West Palm Beach, FL, USA). The surface chemical states of the prepared samples were confirmed by X-ray photoelectron spectroscopy (XPS, Thermo Scientific, Waltham, MA, USA). The structural characteristics of the prepared samples were confirmed by X-ray diffraction (XRD, BRUKER/D2 PHASER, Karlsruhe, Germany) using a CuKα (λ = 0.154 nm) radiation and Raman spectroscopy (Raman, HORIBA, Kyoto, Japan). The XRD patterns were recorded between 10° and 80° at a scanning rate of 5° min^−1^. The surface morphologies of the prepared samples were observed by scanning electron microscopy (SEM, Hitach S-4300, Tokyo, Japan). N_2_ adsorption–desorption isotherms were recorded using a BELSORP measuring instrument (BEL Inc., Toyonaka, Japan) at 77 K to examine the porosities of the prepared samples. All samples were degassed at 160 °C for 10 h before the measurement. The specific surface area was calculated using the Brunauer–Emmett–Teller (BET) equation [36]. The total pore volume was estimated based on the N_2_ adsorption at a P P_0_^−1^ of 0.99. The total micropore volume was calculated by applying the Dubinin–Radushkevich (D-R) equation, and the mesopore volume was determined by subtracting the total micropore volume from the total pore volume [37]. Furthermore, the NLDFT method was used to examine the micropore size distribution [38].

The electrochemical measurements were conducted in three-electrode cells, containing a Pt wire as the counter electrode, Ag/AgCl as the reference electrode, and the prepared samples coated onto nickel foam as the working electrode. For the working electrodes, the samples, carbon black, and polyvinylidene fluoride (PVDF) (80:10:10, *w*/*w*), were mixed and then dispersed in N-methyl-2-pyrrolidone (NMP). Subsequently, the mixture was coated onto a nickel-foam current collector, approximately 1 × 1 cm^2^ in size, which was dried at 100 °C for 12 h. Cyclic voltammetry (CV) and galvanostatic charge–discharge behaviors were examined using an electrochemical analyzer (Iviumstat, Ivium Technologies, The Netherlands) to estimate the electrochemical performance within the potential range of 1.0–0 V in a 6 M KOH solution [39,40,41].

## 3. Results and Discussion

### 3.1. Characterization

**Figure 1:**
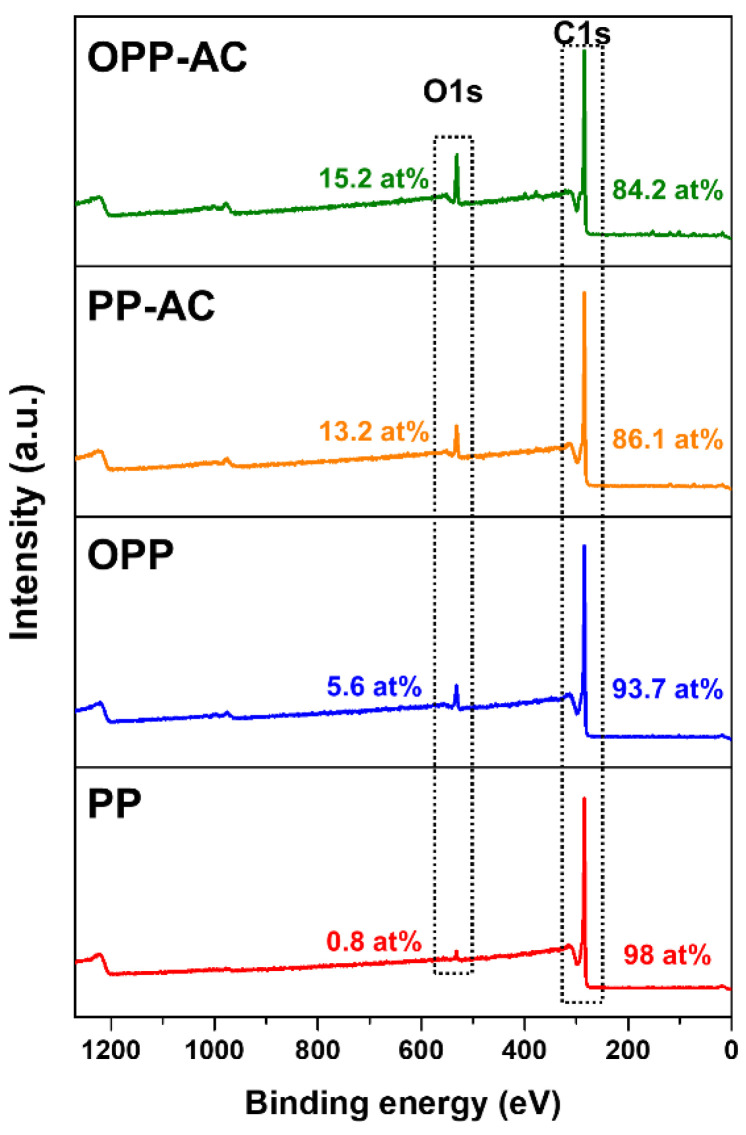
XPS spectra of PP, OPP, PP-AC, and OPP-AC.

The elemental compositions of the prepared samples are listed in Table 1. The oxygen contents of OPP (4.0 wt%) and OPP-AC (11.2 wt%) were found to be higher than those of untreated PP (0.6 wt%) and PP-AC (9.9 wt%) because pre-oxidation introduces oxygen moieties onto pitch molecules [42]. Meanwhile, the hydrogen contents in OPP (4.1 wt%) and OPP-AC (5.5 wt%) were found to be reduced by the pre-oxidation than those of PP (5.3 wt%) and PP-AC (6.0 wt%). Lower H/C atomic ratios of pre-oxidized samples (OPP and OPP-AC) indicated an increased degree of aromaticity [43]. To further explore the surface chemical compositions of the prepared samples, we performed XPS measurements, as shown in Figure 1. All the samples exhibited the C 1s core-level spectrum of graphitic carbon at 285.0 eV, which also has a broad and asymmetric tail towards higher binding energy due to satellite peaks in the range of 288.5–292.5 eV. The peaks of O 1s at 532.1 eV were also observed. The oxygen content of OPP was found to be 5.6 at.%, which is seven-time higher than that of PP (0.8 at.%). This means that the pre-oxidization process provided considerable oxygen moieties onto the PP surfaces. After the chemical activation, the oxygen contents of PP-AC and OPP-AC increased to 13.2 and 15.2 at.%, respectively. These results indicated that KOH activation introduced new oxygen-functional groups onto the carbon surfaces. It is interesting to note that the KOH rapidly entered the carbon frameworks of OPP due to a possible pathway between KOH and surface defects (e.g., oxygen moieties and voids), resulting in a further increase in oxygen content (~15.2 at.%) in OPP-AC [44].

**Figure 2:**
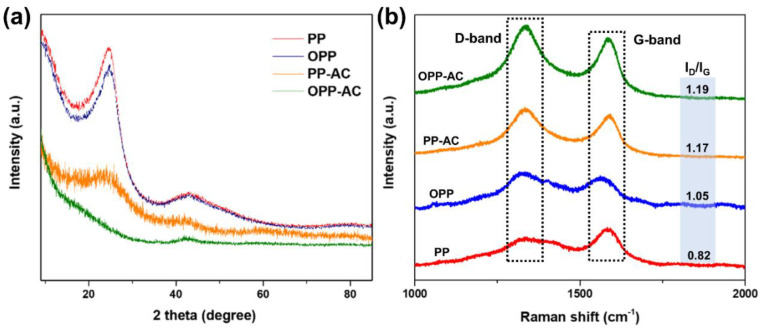
(**a**) X-ray diffractions and (**b**) Raman spectra of PP, OPP, PP-AC, and OPP-AC.

The XRD patterns of the prepared samples are shown in Figure 2a. Two broad diffraction peaks were observed at approximately 25° and 43°, corresponding to the C(002) and C(100) planes, respectively [45]. The C(002) peak of OPP-AC was found to be relatively lower than that of PP-AC, which can be attributed to the disruption of the microcrystalline structure during pre-oxidation, resulting in a reduction of the C(002) peak [46]. The interplanar distance (d) and crystallite thickness (*L_c_*) of samples calculated by Bragg and Scherrer equations [47,48]:

(1) n λ=2d sinθ

(2) $$L_{c}=\frac{K\;\lambda }{\beta \;cos\theta }$$

where n is diffraction order, λ represents the wavelength of the X-rays (0.154 nm), θ indicates the angle of the chosen peak, K is the Scherrer constant, and β is the full width at half-maximum.

As shown in Table 2, the pre-oxidized samples (1.644 nm of OPP and 1.017 nm of OPP-AC) had slightly lower *L_c_* values than their corresponding non-treated samples (1.728 nm of PP and 1.270 nm of PP-AC), respectively. This indicated that pre-oxidation influences the microcrystalline structures, thereby decreasing the crystallinity [49]. The *d_002_* value of the activated carbons (0.371 nm of PP-AC and 0.375 nm of OPP-AC) increased compared to their corresponding precursors (0.360 nm of PP and 0.363 nm of OPP) because of the disordering of the microstructures by chemical activation [50,51]. In addition to XRD, Raman spectroscopy was carried out to observe the microcrystalline properties of the prepared samples (Figure 2b). These results exhibited two characteristic peaks of 1340 and 1579 cm^−1^ corresponding to the D-band and G-band, respectively. The D-band is caused by a defect of graphitic structures, whereas the G-band is associated with the in-plane bond-stretching of sp^2^ hybridized graphitic structure. The relative intensity of D- and G-band (I_D_/I_G_) is often used to estimate the degree of defects in carbon frameworks [52,53]. The I_D_/I_G_ ratios of PP and OPP were 0.82 and 1.05, respectively. The increase is attributable to pre-oxidization, resulting from the introduction of the oxygen-function groups on the carbon surfaces. The increased ratios of PP-AC and OPP-AC showed 1.17 and 1.19, respectively, meaning the highly disordered graphitic structures due to the chemical activation. These are consistent with the XRD results.

**Figure 3:**
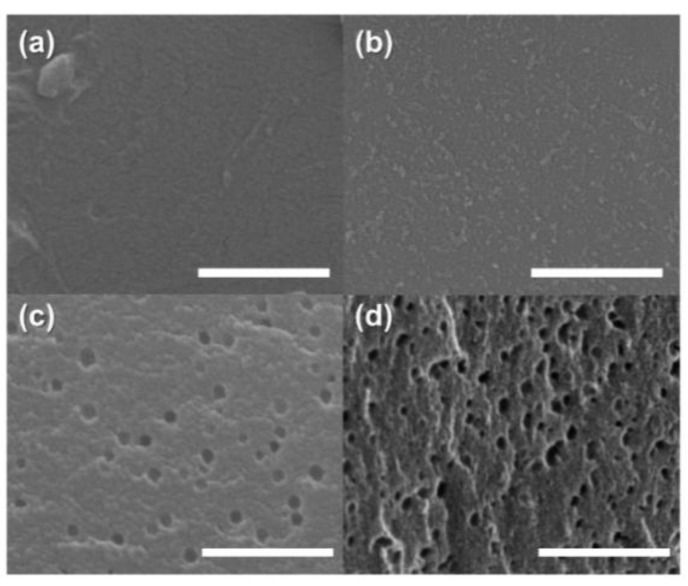
Scanning electron microscopy images of (**a**) PP, (**b**) OPP, (**c**) PP-AC, and (**d**) OPP-AC (scale bar: 500 nm).

The surface morphologies were observed using SEM measurements. As shown in Figure 3a,b, OPP exhibits roughness on its surface, whereas PP has a smooth surface because of the presence of oxygen moieties arising from the pre-oxidation [32]. The external surfaces of the prepared AC samples shown in Figure 3c,d revealed newly developed macropores caused by the external surface decomposition by KOH activation [54]. A higher number of pores were observed in OPP-AC compared to those in PP-AC. This result indicated that the pre-oxidation treatment facilitates the formation of new pores during the activation reaction [55].

**Figure 4:**
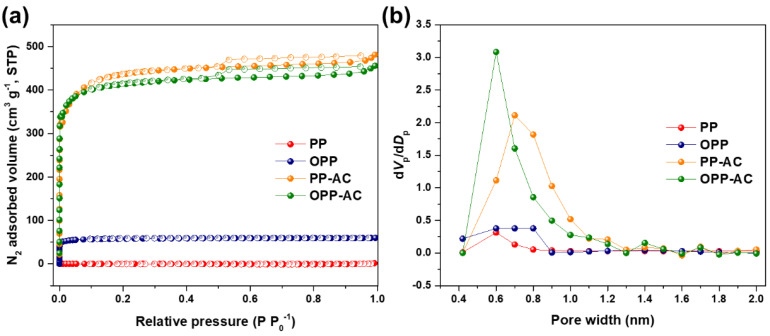
(**a**) N_2_ adsorption-desorption isotherms of the prepared samples at 77 K and (**b**) the pore size distributions using NLDFT method.

In order to investigate the textural properties, N_2_ adsorption analysis was performed at 77 K. As displayed in Figure 4a, all samples except PP have very steep initial N_2_ uptakes at low relative pressures (P P_0_^−1^ < 0.1). The steep rise in these isotherms followed by a sharp knee is due to the capillary filling of micropores. These results also corresponded with the Type-Ι isotherms based on IUPAC classifications, indicating typical microporous carbon [56]. The textural properties of the samples are detailed in Table 3. Micropore volumes of prepared samples were evaluated using the Dubinin–Radushkevich (D-R) equation, which is well-fitted for microporous carbons [37]:

(3) $$W=W_{0}exp\left[-B{\left(\frac{T}{\beta }\right)}^{2}Log^{2}\left(\frac{P}{P_{0}}\right)\right]$$

where W represents the amount of liquid adsorbed at P P_0_^−1^, $W_{0}$ indicates the total amount of adsorbate in the micropores, B is the adsorbent constant, and β is the affinity coefficient.

The equation has a semi-empirical origin and is based on the assumptions of a change in the potential energy between the gas and adsorbed phases and the characteristic energy of a given solid. This equation provides a macroscopic behavior of adsorption loading for a given pressure.

After pre-oxidation, OPP has a specific surface area of 118 m^2^ g^−1^, which is approximately 60 times higher than that of PP (2 m^2^ g^−1^). OPP also has a much higher total pore volume (0.12 cm^3^ g^−1^) and micropore volume ratio to total pore volume (58%) than those of PP (0.03 cm^3^ g^−1^ and 33%), as shown in Table 3. These results clearly indicated that pre-oxidation is crucial in improving the textural properties of PP. Notably, PP-AC exhibited a higher specific surface area (1327 m^2^ g^−1^) and total pore volume (0.74 cm^3^ g^−1^) than that of OPP-AC (1246 m^2^ g^−1^ and 0.70 cm^3^ g^−1^). However, PP-AC and OPP-AC have an almost similar value of the micropore volumes (approximately 0.59 cm^3^ g^−1^), and the pore size distribution of OPP-AC was much narrower than that of PP-AC, as shown in Figure 4b. This indicated that pre-oxidation promotes a highly developed micropore structure [57]. The pore size distributions also show that OPP-AC had a higher peak at smaller pore width of 0.6 nm than that of PP-AC (0.7 nm). These results suggested that pre-oxidation provided much smaller micropores in the carbon frameworks.

### 3.2. Electrochemical Performances of the Pitch-Based Activated Carbon Samples

**Figure 5:**
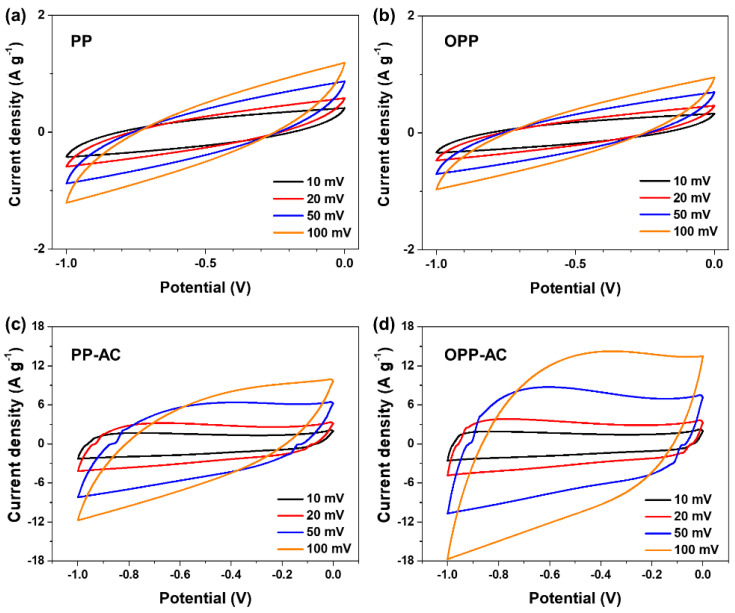
Cyclic voltammetry curves of (**a**) PP, (**b**) OPP, (**c**) PP-AC, and (**d**) OPP-AC at scan rates from 10 to 100 mV s^−^^1^ in 6 M KOH electrolyte.

As shown in Figure 5, the electrochemical performance of prepared samples was investigated based on cyclic voltammetry (CV) curves recorded in the range of 10–100 mV s^−1^ in a 6 M KOH electrolyte solution. The CV curves of all the samples exhibited a quasi-rectangular shape, indicating ideal capacitance behaviors [58]. Moreover, all recorded CVs retained their original shapes upon increasing the scan rate from 10 to 100 mV s^−1^. These results suggest that all the samples exhibited good rate capability and high stability across the scan rate range [59]. The CV curve area of OPP-AC was found to be larger than that of PP-AC, indicating its higher electrochemical performance compared to that of PP-AC [60]. This can be attributed to the nano-channels formed by micropores due to pre-oxidation, resulting in rapid penetration of the electrolyte ions [61].

**Figure 6:**
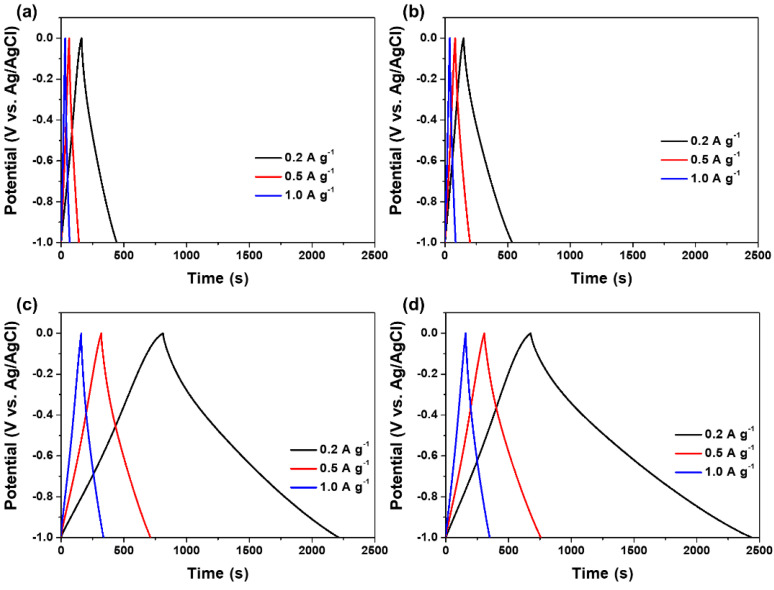
Charge–discharge curves of (**a**) PP, (**b**) OPP, (**c**) PP-AC, and (**d**) OPP-AC with different current densities from 0.2 to 1.0 A g^−1^ in 6 M KOH electrolyte.

The galvanostatic charge–discharge curves of the prepared samples were obtained as functions of current density, as shown in Figure 6. The charge–discharge duration of the prepared samples increased with a decrease in current density from 1.0 to 0.2 A g^−1^. The charge–discharge curves of the prepared samples exhibited an almost triangular shape, indicating their excellent rate capabilities and reversibility [62,63]. A longer discharging time than the charging time observed in OPP-AC could be attributed to the oxygen moieties on the electrode surface, resulting from the interactions between the electrode and electrolyte [64].

The specific capacitances ($C_{sp}$) of the prepared samples are listed in Table 4. These are calculated based on the galvanostatic charge–discharge method using the following equation [65]:

(4) $$C_{sp}=\frac{I\Delta t}{m\Delta V}$$

where $C_{sp}$ represents the specific capacitance (F g^−1^), I indicates the current, Δt is the discharge time, m represents the mass of the active material, and ΔV is the potential window.

**Figure 7:**
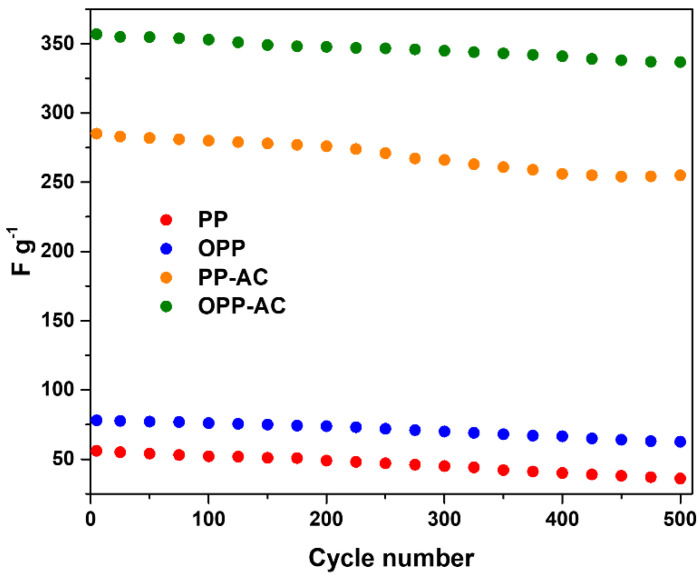
Cycling performance of the prepared samples at a current density of 0.2 A g^−^^1^ in 6 M KOH electrolyte.

The estimated $C_{sp}$ values of OPP-AC and PP-AC were found to be 357 and 285 F g^−1^, respectively, at a current density of 0.2 A g^−1^. The higher $C_{sp}$ value of OPP-AC is because of the well-developed micropores, which promote efficient ionic transfers between the electrode and electrolyte [66]. The cyclic stability of the prepared samples was also evaluated at a current density of 0.2 A g^−1^ in 6 M KOH electrolyte, as shown in Figure 7. The specific capacitances of PP-AC and OPP-AC were maintained at more than 89% and 94%, respectively, for the initial capacitances after 500 cycles. The OPP-AC showed superior cycling stability, resulting from oxygen moieties due to the higher wettability [67].

Several studies also reported this interesting phenomenon of higher specific capacitance even though the electrodes exhibited a quite low specific surface area. A possesses of the efficient pore sizes of electrode materials should facilitate the electrolyte ions to access the pores, enhancing the electrochemical performance of EDLCs. You et al. prepared lignin-based activated carbon fibers (LACFs) by electrospinning and subsequent steam activation for electrochemical behaviors. They reported that LACF-105 and LACF-180 (here, the numbers at the end of the specimen refer to the different amounts of water consumed during the steam activation) exhibited specific surface areas of 2185 and 2447 m^2^ g^−1^, respectively. The pore size distribution of LACF-105 was predominant in the pore size range of 0.5–0.14 nm, whereas that of LACF-180 appeared in the range of 1.4–3.0 nm. Additionally, the specific capacitance was found to be 133.3 F g^−1^ at the current density of 1 A g^−1^ in LACF-105, which is approximately two times higher than that of 63.5 F g^−1^ in LACF-180. It is clearly indicated that the smaller pore sizes that emerged from the steam activation play a key role in enhancing the EDLCs’ performance [68]. Tian et al. investigated flute type-microporous activated carbons (FTMAC) from cotton stalk via KOH activation as a function of the KOH agent amount for electrochemical behaviors. The weight ratios of KOH to the carbonized cotton stalk were denoted at the end of FTMAC. FTMAC-4 showed the highest specific capacitance of 254 F g^−1^ at a current density of 0.2 A g^−1^, although FTMAC-4 exhibited a lower specific surface area of 1964 m^2^ g^−1^ than that of FTMAC-5 of 2251 m^2^ g^−1^ [69]. Additionally, Chmiola et al. prepared carbide-derived carbons (CDC) to study the effect of the pore sizes on the electrochemical behaviors. This result showed that the specific surface area and specific capacitance do not have a linear relationship. They demonstrated that micropores smaller than 1 nm have significantly influenced the improvement of specific capacitances [14]. In the case of graphene materials for EDLCs, He et al. synthesized porous graphene nanosheets (CGNSs) from petroleum pitch using KOH activation as a function of activation temperature. The specific surface area of 2132 m^2^ g^−1^ in the CGNS_3-1073_ was found to be slightly lower than that of 2216 m^2^ g^−1^ in the CGNS_3-1173_. However, the electrochemical performances, including specific capacitance and cycle stability of the CGNS_3-1073_, were much higher than those of CGNS_3-1173_. This can be attributable to the much smaller micropores formed in the CGNSs, facilitating the ions to access the electrode surfaces [28]. Thus, as observed, it is suggested that the designing of carbon materials with efficient pore sizes is critical to enhancing electrochemical performances. In addition, we listed several reports of PP-based porous materials for EDLCs in order to compare our results with others [23,29,70,71,72,73,74]. Table 5 lists the specific capacitances of PP-based EDLCs and the specific surface areas at different evaluation conditions. It was found that our result exhibited among the highest in the comparison, even though it had a quite lower specific surface area. A linear relationship between the specific surface area and the specific capacitances is also not shown [75,76].

## 4. Conclusions

In this study, the pre-oxidized petroleum pitch (PP)-derived activated carbon (OPP-AC) was successfully prepared for use in electric double-layer capacitors (EDLCs). These experimental results revealed that OPP-AC and PP-AC exhibit specific capacitances of 357 and 285 F g^−1^, respectively, at a current density of 0.2 A g^−1^. These results can be attributed to the narrower pore size distribution and the efficient pore sizes of OPP-AC. Therefore, this study demonstrated that the pre-oxidation process affects the development of micropores in the OPP-AC, providing efficient nano-channels to enhance electrochemical performance. Consequently, pre-oxidation is a promising strategy to ensure the high performance of EDLCs through effective micropore generation.

## Figures and Tables

**Table 1:** Elemental analysis of PP, OPP, PP-AC, and OPP-AC.

Specimens	C (wt%)	N (wt%)	H (wt%)	S (wt%)	O (wt%)	H/C
PP	89.0	0.1	5.3	0.5	0.6	0.7
OPP	88.5	0.1	4.1	0.5	4.0	0.6
PP-AC	83.7	0.1	6.0	0.3	9.9	0.9
OPP-AC	82.9	0.1	5.5	0.3	11.2	0.8

**Table 2:** Microcrystalline structural parameters of PP, OPP, PP-AC, and OPP-AC.

Specimens	*d_002_* (nm) ^a^	*L_c_* (nm) ^b^
PP	0.360	1.728
OPP	0.363	1.644
PP-AC	0.371	1.270
OPP-AC	0.375	1.017

^a^ Interplanar distance (nm) determined from Bragg’s equation. ^b^ Crystallite thickness (nm) determined from Scherrer’s equation.

**Table 3:** Porous structural parameters of PP, OPP, PP-AC, and OPP-AC.

Specimens	S_BET_^a^ (m^2^ g^−1^)	V_total_^b^ (cm^3^ g^−1^)	V_micro_^c^ (cm^3^ g^−1^)	V_meso_^d^ (cm^3^ g^−1^)	V_micro_/V_total_^e^ (%)
PP	2	0.03	0.01	0.02	33
OPP	118	0.12	0.07	0.05	58
PP-AC	1327	0.74	0.59	0.15	80
OPP-AC	1246	0.70	0.59	0.11	84

^a^ S_BET_: specific surface area computed using BET equation at a relative pressure range of 0.001–0.01. ^b^ V_total_: total pore volume determined at P P_0_^−1^ of 0.99. ^c^ V_micro_: micropore (0–2 nm) volume determined from the D-R method. ^d^ V_meso_: mesopore (2–50 nm) volume determined by subtracting the micropore volume from the total pore volume. ^e^ V_micro_/V_total_: micropore volume ratio to total pore volume (%).

**Table 4:** Specific capacitances of PP, OPP, PP-AC, and OPP-AC.

Specimens	0.2 A g^−1^	0.5 A g^−1^	1.0 A g^−1^
PP	56	39	36
OPP	78	63	46
PP-AC	285	200	182
OPP-AC	357	228	195

**Table 5:** Comparison of specific capacitances of petroleum pitch-based EDLCs.

Specimens	Electrolyte	Current Density (A g^−1^)	Specific Surface Area (m^2^ g^−1^)	Specific Capacitance (F g^−1^)	Ref
OPP-AC	6 M KOH	0.2 (1.0)	1246	357 (195)	This work
PP/Polypyrrole	1 M Na_2_SO_4_	1.0	602	82	[23]
PP/MnO_2_	0.5 M Na_2_SO_4_	0.5	787	92	[29]
PP/Coal tar pitch	1 M TEABF_4_	1.0	2967	132	[70]
PP-based ACs	6 M KOH	0.05	2964	300	[71]
PP-based ACs	6 M KOH	0.05	3516	320	[72]
PP-based ACs	6 M KOH	0.05	1922	293	[73]
PP-based ACs	6 M KOH	0.05	2646	296	[74]

## Data Availability

Not applicable.
